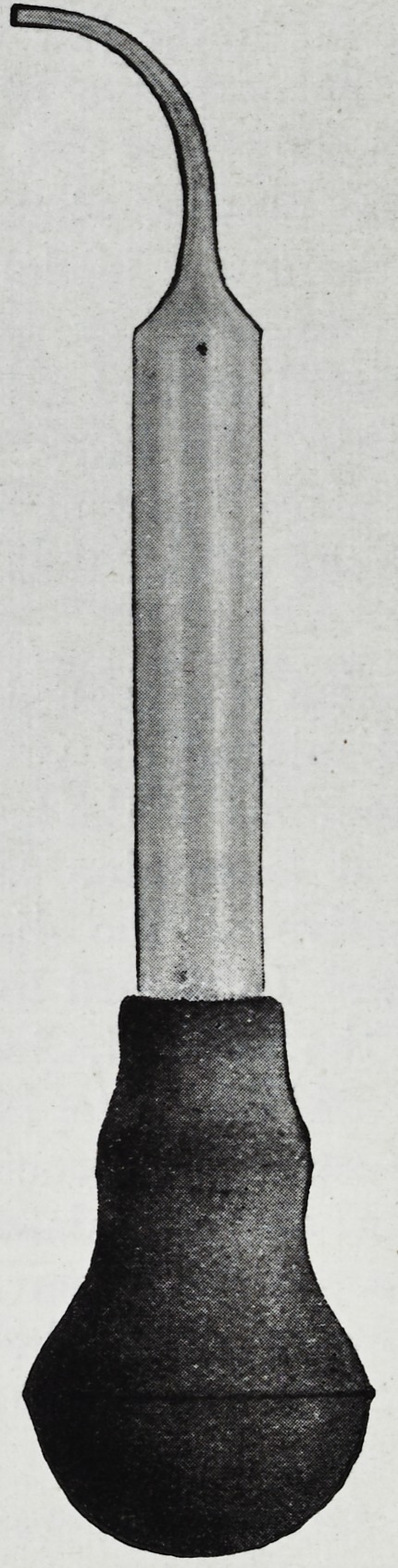# The Glyco-Thymoline Applicator and Its Use in Pyorrhoea, Abscessed Conditions and General Oral Hygiene

**Published:** 1904-04

**Authors:** C. T. Dahlin

**Affiliations:** Elgin, Ill.


### THE GLYCO-THYMOLINE APPLICATOR AND ITS USE IN PYORRHOEA, ABSCESSED CONDITIONS AND GENERAL ORAL HYGIENE.

By C. T. Dahlin, D.D.S., Elgin, Ill.

In the treatment of Pyorrhoea Alveolaris, Alveolar Abscesses and ulcerated conditions, we are frequently called upon to direct home treatment, supplemented to that at the office.

An inexpensive little applicator has been devised by the Kress and Owen Company for the application of Glyco-Thymoline by the patient by inserting the fine point of this miniature syringe between the tooth and gums and socket completely filled with the antiseptic solution. This treatment is to be done two or three times a day and the last thing before retiring, at which time the cavity is kept under the influence of the treatment for hours while the mouth, gums and teeth are in a state of comparative quiet. The gratifying results which are obtained in these troublesome conditions by the use of this little instrument will justify any dentist in adopting it.

There is another department of oral sanitation for which I wish to commend the applicator, namely, the proper cleansing of the various forms of bridge work. The varying conditions that confront us in this work are many and despite all ingenuity of construction, bridges are often unsatisfactory because of the difficulty and sometimes impossibility of giving to them proper care. And this is not always the fault of the patient, who may be conscientious in his efforts, but rather to the dentist in not providing the means whereby he can accomplish the end sought after. It may be said that 90 per cent. of all bridges are in a greater or lesser degree unsanitary when nothing but the brush is used in maintaining its cleanliness.

It is here that the "applicator" will serve an excellent purpose in forcing a stream of dilute Glyco-Thymoline into the interstices and obscure places of the bridge appliance. It will be readily seen how great will be the benefit; to the patient for obvious reasons, and to the practitioner in that success will attend his efforts where otherwise failure and disrepute must certainly follow.

Under this method we maintain an aseptic condition of the mouth which makes our bridge patients a constant joy to us rather than as frequently occurs, they are the cause of serious difficulty and produce almost constant fear for their future. —*Dental Times.*

## Figures and Tables

**Figure f1:**